# Clinical and Parasitological Characteristics of *Plasmodium vivax *Malaria in Malaria-Naïve Patients: A Review of Malaria Fever Therapy in Patients with Schizophrenia and Neurosyphilis during the 1950s and 1960s in Vienna, Austria

**DOI:** 10.4269/ajtmh.24-0828

**Published:** 2025-09-30

**Authors:** Simone Wolff, Rosa Maria Kainz, Dietrich Reimold, Heimo Lagler, Johannes Mischlinger, Michael Ramharter

**Affiliations:** ^1^ Bernhard Nocht Institute for Tropical Medicine and I. Dep. of Medicine, University Medical Center Hamburg-Eppendorf, Hamburg, Germany;; ^2^ Clinical Division for Infectious Diseases and Tropical Medicine, Medical University of Vienna, Vienna, Austria;; ^3^ German Center for Infection Research, Partner Site Hamburg-Lübeck-Borstel-Riems, Hamburg, Germany;; ^4^ Centre de Recherches Médicales de Lambaréné, Lambarene, Gabon

## Abstract

On the basis of the findings of Austrian psychiatrist Julius Wagner-Jauregg, malaria fever therapy became the standard treatment for end-stage syphilis associated with generalized paralysis in the early 20th century. The parasitological and clinical features of iatrogenically induced *Plasmodium vivax* malaria in patients with schizophrenia and neurosyphilis during the 1950s and 1960s in Vienna, Austria, are described in the current study. All patients treated for schizophrenia or neurosyphilis at the Department of Psychiatry of the General Hospital of Vienna between 1951 and 1969 who underwent malaria fever therapy were analyzed regarding the parasitological and clinical characteristics of induced malaria. A total of 322 patients who underwent malaria fever therapy were included in the analysis (schizophrenia: *n* = 147; neurosyphilis: *n* = 175). The route of inoculation was mainly intravenous, and the dose varied between 4 and 8 mL of blood. The first fever peaks appeared ∼7 days post-inoculation. Temperature increased over time in consecutive fever paroxysms, whereas the afebrile time interval between fever peaks shortened progressively from 41 to 31 hours. After a mean of 5–6 fever peaks, all patients received standard antimalarial therapy with quinine monotherapy or combination therapy. These data reveal that the extra-hepatic incubation period of *P. vivax* is ∼7 days after intravenous inoculation. The current study reveals a surprisingly short periodicity between fever paroxysms, shedding light on the natural course of infection. The evaluation of historic patient data from malaria fever therapy provides a unique opportunity to study the clinical and parasitological features of untreated malaria.

## INTRODUCTION

Malaria is a life-threatening parasitic disease and one of the greatest challenges to global health. According to the WHO, almost half of the world’s population was at risk of contracting malaria in 2020, with *Plasmodium falciparum* and *Plasmodium vivax* being the predominant species causing malaria.^1^ Most cases and deaths occur in the tropics and subtropics, especially sub-Saharan Africa, but malaria was also widespread in northern Europe and North America, particularly until the end of World War II.^2^ Control interventions, such as the implementation of national elimination programs, antimalarial chemotherapy, and the use of insecticides, led to the elimination of malaria in Europe by 1978.^3^

In addition to naturally acquired *Plasmodium* infections, controlled human malaria infection became a medical intervention in Western medicine. In this context, the Austrian Julius Wagner-Jauregg is considered a pioneer of malaria fever therapy.^4^^–^^6^ On the basis of his observation that febrile diseases in patients with end-stage syphilis-associated generalized paralysis (general paralysis of the insane [GPI]) sometimes led to an improvement in their mental status, he began to conduct experiments with iatrogenically induced fever in patients suffering from psychosis and GPI in 1894. A link between GPI and syphilis infection had not yet been identified at that time; it was later proven by the bacteriologist Hideyo Noguchi in 1913.^7^

In 1919, Wagner-Jauregg published his first results in the publication “*On the Impact of Malaria on the Paralysis of the Insane*.” Subsequently, malaria therapy became the standard treatment for GPI despite a mortality rate of 4–20%.^8^^,^^9^ Wagner-Jauregg was awarded the Nobel Prize in Medicine in 1927 for this discovery, making him the first and only psychiatrist to receive it to date.^10^

In subsequent decades, controlled human malaria infections were used to treat various other diseases, such as psychosis, depression, and other behavioral characteristics perceived as abnormal, including alcoholism, homosexuality, and so-called antisocial behavior. Induced malaria infections also played a role in the context of drug and vaccine research.^11^ Most recently, malaria fever therapy has reemerged in discussions of potential treatment strategies for different diseases.^12^^,^^13^

With the introduction of penicillin and its effectiveness against *Treponema pallidum*, malaria therapy became increasingly obsolete after World War II. However, in German-speaking countries, particularly in Vienna, malaria fever therapy continued until the late 1960s. On the one hand, this might be explained by physicians being influenced by local tradition and following Julius Wagner-Jauregg’s legacy, and on the other hand, it might be explained by the fact that a malaria strain for the iatrogenic inoculation of *P. vivax* was available in Vienna longer than it was anywhere else.^14^ In addition, malaria fever therapy was used as an established method to treat conditions considered untreatable at that time. Because no mosquito-based infection cycles were available in Austria, actively infected patients were required to maintain this therapeutic method. The often-used combination of penicillin and malaria fever therapy was also based on the idea that hyperthermia improves the permeability of the blood–brain barrier, thereby increasing the antibiotic’s penetration. It was also based on the probable activity of increased temperature against *T. pallidum* infection.^15^

For iatrogenically induced malaria infections, patient blood infected with *P. vivax* was primarily used instead of the more virulent *P. falciparum* to reduce the risk of a deleterious outcome from the induced malaria itself while still ensuring high fever peaks.^16^^,^^17^ As part of malaria fever therapy, blood was taken from patients suffering from acute vivax malaria during a fever episode and before starting antimalarial therapy; it was then inoculated via an intramuscular or intravenous (IV) route to a patient with neurosyphilis or other indications for malaria fever therapy.

In the case of syphilis patients, transmission most commonly occurred from donors without syphilis; however, cases of blood transfer from one syphilis patient to another were documented. For non-syphilis patients, a chain of non-syphilis-infected blood was maintained and presumably had already been in place since the 1920s in Vienna. When the continued chain of malaria blood carriers was interrupted in Vienna in 1959, blood was obtained from the Tropical Medicine Institutes in Hamburg and Amsterdam.^14^

However, the injected blood contained only erythrocytic developmental stages of the pathogen and no pre-erythrocytic developmental stages. Unlike naturally infected patients, patients infected via IV inoculation do not harbor any dormant liver-stage hypnozoites that may cause relapses of malaria. Antimalarial therapy with standard blood schizontocidal drugs—at that time, most commonly quinine in combination with atebrin (i.e., mepacrine) or chloroquine—was therefore sufficient for a radical cure.

In addition to elucidating historic therapeutic approaches, the analysis of these data constitutes a unique opportunity in medical parasitology to study the natural course of untreated *P. vivax* infection—a feature that cannot be studied today because of ethical limitations. In the present study, the archived files of all medical records of patients treated with controlled malaria fever therapy at the Department for Psychiatry, General Hospital of Vienna, between 1951 and 1969 were analyzed. To obtain a coherent patient population, data from patients who were treated for GPI or schizophrenia—the two most common indications for malaria fever therapy in those times—were included.

The aim of this retrospective study was to describe the parasitological and clinical features of induced malaria infection with *P. vivax*, as well as the natural course of untreated disease until the administration of antimalarial treatment in malaria-naïve subjects.

## MATERIALS AND METHODS

Archived medical records of patients treated with malaria fever therapy between 1951 and 1969 at the adult wards of the Department of Psychiatry and Psychotherapy, General Hospital of Vienna, were analyzed. The period corresponds to the final two decades when malaria fever therapy was used at the Department of Psychiatry at the University Hospital of Vienna. Although malaria fever therapy was stopped shortly after penicillin was made available in most treatment centers worldwide, Vienna remained an exception and continued this medical practice until the end of the 1960s.

Only patients with a primary diagnosis of schizophrenia or neurosyphilis, as an indication for malaria fever therapy, were included in the present study. There was a plethora of other medical indications in a heterogeneous patient population. All patients with other primary diagnoses, as well as those with incomplete clinical documentation, were excluded from the analysis. None of the included patients had a known history of previous malaria infection.

The following diagnoses were summarized as a diagnosis of schizophrenia: *Hebephrenie*, *Propfhebephrenie*, *paranoide Schizophrenie*, *alte katatonische Schizophrenie*, *alte Schizophrenie*, *Schizoider Defekt*, and *Schizophrenie*. The diagnosis of syphilis infection was considered confirmed when a cerebrospinal fluid puncture and a Wassermann reaction were performed, revealing results indicative of syphilis. In the Wassermann reaction, blood or cerebrospinal fluid was exposed to cardiolipin, leading to an antibody reaction. Although it is not specific, the Wassermann reaction was widely used as an antibody test for syphilis and has since been replaced by newer nontreponemal tests, such as rapid plasma reagin and venereal disease research laboratory. No further data are available about the clinical definitions of the respective diagnoses.

In addition to basic demographic data, various other parameters were recorded and evaluated from the medical records, including additional therapies, inoculation dose, inoculation route, time from inoculation to fever peak, the time interval between successive fever peaks, the number of fever peaks, the highest body temperature at each fever peak, and the medication used for subsequent antimalarial therapy.

Fever is generally defined as a body temperature of 38.5°C or higher, whereas temperatures below 38.5°C are considered subfebrile. In the patient files evaluated in the current study, the baseline was specified with a normal body temperature of 37°C. Accordingly, a dichotomized distinction was made between afebrile (baseline 37°C or below) and febrile. It was not noted in the records where the temperature was measured. Because the exact time of inoculation was not documented in the patient files, 6 am was chosen as the inoculation time for all patients, according to the usual daily medical routine at that time.

In addition to malaria fever therapy, other therapeutic methods were used, as documented in the patient files. Complementary therapies included sequential provocation therapy with typhoid vaccine (attenuated *Salmonella typhi* pathogens), insulin shock therapy, electroshock therapy, and additional penicillin, or combinations of different additional therapies.

With regards to laboratory investigations, parameters such as white blood cell count, hemoglobin, and erythrocyte count were recorded before or after therapy. For the quantification of hemoglobin, Sahli’s method was used, and the color coefficient was subsequently calculated. Data analysis was mainly performed using descriptive statistics. For statistical analysis, two-sided *t*-tests for unpaired samples were used; a *P*-value <0.05 was defined as significant.

The present study was conducted with a focus on the parasitological and clinical aspects of malaria infections induced by treating physicians, without assessing the treatment outcomes of psychiatric conditions or examining the medico-legal or ethical aspects of this medical practice. The historical reappraisal of the medical use of malaria fever therapy at the Department of Psychiatry has been thoroughly assessed and is summarized in a scientific report.^14^

## RESULTS

### Study population.

A total of 869 patients who were iatrogenically infected with *P. vivax* at the General Hospital in Vienna between 1951 and 1969 were identified in the archive. In 158 subjects, the main diagnosis was schizophrenia, whereas in 175 subjects, neurosyphilis was the indication for treatment with malaria fever therapy. In both diagnostic groups, the proportion of men was predominant (schizophrenia: *n* = 154/158, 97% male; neurosyphilis: *n* = 118/175, 67% male). The mean age in the group suffering from schizophrenia was 23.8 ± 6.7 years, whereas in the group suffering from neurosyphilis, it was 44.8 ± 9.8 years. Because the focus of this study was primarily on the parasitological and clinical aspects of iatrogenic *P. vivax* infection in malaria-naïve patients, only complete records of temperature evolution over time have been included in the analysis. In both groups, the majority of cases were inoculated via an IV route. The inoculation doses varied between 4 and 8 mL, with most subjects being infected with 4 mL of blood containing *P. vivax* blood-stage parasites, regardless of whether they received IV or intramuscular (IM) inoculation. The medical records did not provide any reason for the decision regarding the respective therapy dose or inoculation route. In the present cohort, one patient suffering from schizophrenia died during inpatient treatment. The medical records reveal no clear cause of death while indicating that the patient had been inoculated with *P. vivax*-infected blood and had subsequently received antimalarial therapy before succumbing during the hospital stay.

### Fever paroxysms.

**Figure 1.**
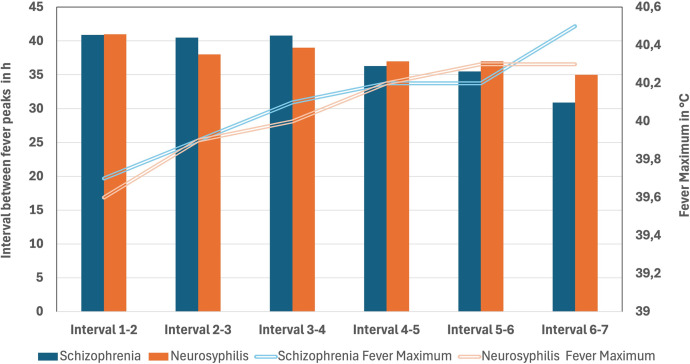
Illustration of the shortening of the mean time span between fever peaks, which correlates with the total number of fever periods in both groups, along with an increase in the mean maximum body temperature associated with the increasing number of fever periods.

In the group of schizophrenia patients, at least one fever peak was documented in 139/147 subjects (95%); in eight patients, no reaction to *P. vivax* inoculation could be identified. On average, the incubation time until the first fever peak was 7.0 ± 2.7 days. In the group of neurosyphilis subjects, the time span from inoculation to the first fever episode was on average 7.7 ± 3.1 days. With regard to the total number of documented fever periods, an average of 5.4 ± 1.5 fever periods was recorded in the schizophrenia group, and an average of 6.3 ± 2.1 fever periods was recorded in the neurosyphilis group. In the analysis of the time intervals between the fever peaks, a shortening of the mean time span between the fever peaks was observed to correlate with the number of total fever periods in both groups (Table 1; Figure 1). The interval between two fever episodes for individuals who experienced seven fever episodes was shorter throughout the observation period than for the overall cohort (i.e., 30.5 hours [interquartile range (IQR): 24–42] versus 39 hours [IQR: 27–45]). In contrast, an increase was observed in the mean maximum body temperature measured during the consecutive fever paroxysms.

**Table 1:** Clinical and parasitological characteristics of patients diagnosed with schizophrenia or neurosyphilis who underwent malaria fever therapy between 1951 and 1969 in Vienna

Category	Schizophrenia	Neurosyphilis
Diagnosis (*n*)	158	175
Sex (*n*)	154 M/4 F	118 M/57 F
Number of records with complete data for temperature evolution (*n*)	147 (145 M/2 F)	82 (62 M/20 F)
Mean age* ±* SD (range, years)	23.8* ±* 6.7 (13–43)	44.8* ±* 9.8 (11–69)
Inoculation with *P. vivax* (*n*)	147	175
Inoculation route (*n*)	IV: 117 (115 M/2 F)	IV: 153 (102 M/51 F)
	IM: 30 (M)	IM: 11 (9 M/2 F)
		Unknown: 1
		Transferred to other hospitals: 10
Inoculation dose (*n*)		
4 mL	132 (130 M/2 F)	137 (89 M/48 F)
5 mL	6 (M)	12 (9 M/3 F)
6 mL	8 (M)	15 (14 M/1 F)
8 mL	1 (M)	1 (M)
Number of fever periods (*n*; mean* ±* SD)	5.4* ±* 1.5	6.3* ±* 2.1
Interval: inoculation to first fever period (days; mean* ±* SD)	7.0* ±* 2.7	7.7* ±* 3.1
	11.6* ±* 3.5 (subgroup typhoid provocation)	9.7* ±* 3.8 (subgroup typhoid provocation)
Interval: First/second fever peak (hours; mean* ±* SD)	41* ±* 18	41* ±* 22
	67* ±* 47 (subgroup typhoid provocation)	57* ±* 39 (subgroup typhoid provocation)
Interval: Sixth/seventh fever peak (hours; mean* ±* SD)	31* ±* 11	35* ±* 10
	37* ±* 12 (subgroup typhoid provocation)	40* ±* 8 (subgroup typhoid provocation)
Maximum body temperature per fever episode		
First fever episode (°C, mean* ±* SD, range)	39.7* ±* 0.5 (38.6–40.8)	39.6* ±* 0.6 (38.3–41.0)
Second fever episode (°C, mean* ±* SD, range)	39.9* ±* 0.5 (38.5–43.0)	39.9* ±* 0.5 (38.5–41.0)
Third fever episode (°C, mean* ±* SD, range)	40.1* ±* 0.4 (38.5–41.2)	40.0* ±* 0.6 (38.3–41.0)
Fourth fever episode (°C, mean* ±* SD, range)	40.2* ±* 0.4 (39.4–41.2)	40.2* ±* 0.5 (38.8–42.0)
Fifth fever episode (°C, mean* ±* SD, range)	40.2* ±* 0.6 (38.4–42.0)	40.3* ±* 0.5 (38.5–41.1)
Sixth fever episode (°C, mean* ±* SD, range)	40.2* ±* 0.5 (38.7–41.0)	40.3* ±* 0.5 (39.0–41.1)
Seventh fever episode (°C, mean* ±* SD, range)	40.1* ±* 0.5 (39.0–41.0)	40.2* ±* 0.5 (38.7–41.2)
Ancillary therapy (*n*)	147	175
Malaria fever therapy only	93	77
Malaria + typhoid provocation	11	6
Malaria + electroshock	29	3
Malaria + typhoid + electroshock	3	–
Malaria + insulin shock	3	–
Malaria + insulin shock + electroshock	7	–
Malaria + insulin shock + electroshock + typhoid	1	–
Malaria + penicillin	–	65
Malaria + penicillin + electroshock	–	6
Malaria + penicillin + typhoid	–	15
Malaria + penicillin + electroshock + insulin shock	–	1
Malaria + penicillin + typhoid + antabus	–	1
Malaria + penicillin + cardiazole shock	–	1
Antimalarial therapy	146	82
Quinine monotherapy	2	5
Quinine + atebrine	118	59
Quinine + chloroquine	26	17
Plasmochin + atebrine	–	1
Laboratory results		
Number of patients with available blood tests	15	43
Before malaria fever therapy (*n*)	5	36
After malaria fever therapy (*n*)	10	10
Before and after malaria fever therapy (*n*)	–	3
Erythrocyte count (10^12^/L; mean* ±* SD; before/after malaria fever therapy)	4.35* ±* 0.41/3.96* ±* 0.28	4.14* ±* 0.41/3.67* ±* 0.53
Leukocyte count (10^9^/L; mean* ±* SD; before/after malaria fever therapy)	4.61* ±* 0.76/7.48* ±* 3.14	6.13* ±* 1.64/6.62* ±* 2.05
Hemoglobin (g/dL; converted from Sahli’s method; mean* ±* SD; before/after malaria fever therapy)	12.2* ±* 0.8/11.4* ±* 0.8	12.0* ±* 1.2/10.9* ±* 1.3

F = female; M = male; *P. vivax* = *Plasmodium vivax*; SD = standard deviation.

When comparing the two inoculation routes, there was a statistically significantly longer time between inoculation and the first fever episode in the intramuscularly infected subjects (schizophrenia group: 9.1 ± 3.0 days IM versus 6.6 ± 2.3 days IV; *P* <0.0001). Regarding the increase in the maximum fever temperature up to the seventh fever episode (IM route) or fourth fever episode (IV route), there were slightly higher temperatures on average in the IM group.

All patients in the schizophrenia and neurosyphilis groups received antimalarial therapy to stop the malaria fever, as shown in Table 1. For this purpose, quinine and atabrine were used until this practice was changed to the use of quinine and chloroquine in the 1960s.

### Ancillary therapy.

In addition to malaria fever therapy, other therapeutic methods were applied in both groups of subjects, as shown in Table 1. All complementary treatment procedures have historically been used to treat psychosis; they were apparently used in this cohort because of an insufficient treatment response to the underlying psychiatric condition.

### Typhoid provocation therapy.

In the schizophrenia group, typhoid provocation therapy was performed in 15 male subjects; in the neurosyphilis group, the typhoid inoculation was administered to provoke fever in a total of 22 subjects. Injections of attenuated *S. typhi* pathogens were used to provoke fever in subjects who did not react to the previous malaria inoculation. Therefore, and not surprisingly, in both study groups, a significantly longer time span was found from inoculation of the malaria pathogen to the first fever peak compared with the subjects without typhoid provocation. Additionally, in subjects with typhoid provocation, there was a comparable trend toward an increasing maximum temperature up to approximately the sixth or seventh fever episode and a decrease in the time interval between individual fever peaks with an increasing number of fever episodes.

The analysis of the available laboratory data was limited by a substantial proportion of missing data. Daily blood smears were performed according to the medical records; however, no parasitaemia results were documented in any of the medical files.

In the schizophrenia group, blood tests were recorded in a total of 15/147 patients. None of the patients had more than one blood test performed. For the neurosyphilis group, results from 36 blood samples taken before malaria fever therapy and 10 samples taken after malaria fever therapy were available. In a total of three patients, laboratory tests were performed both before and after malaria fever therapy, and in one patient, tests were performed before and during malaria fever therapy. Both groups showed comparable results with a decrease in the erythrocyte count and hemoglobin and an increase in the leukocyte count after malaria fever therapy compared with the results available before malaria fever therapy (Table 1).

## DISCUSSION

The present study is focused on parasitological and clinical characteristics of iatrogenic *P. vivax* infection in malaria-naïve patients diagnosed with GPI or schizophrenia in Vienna in the 1950s and 1960s. Overall, both patient groups exhibited substantial heterogeneity in terms of age and, particularly, the representation of sex within each group. Schizophrenia patients were significantly younger than neurosyphilis patients, with a mean age of 24 years, and the vast majority of patients were male. One may speculate about the reasons for the unequal sex distribution. According to current data, men generally have a higher risk of developing schizophrenia than women, with a risk ratio of 1.4:1.^18^ In addition, men tend to have an earlier age of onset of schizophrenia and worse outcomes.^19^^,^^20^

In contrast, the diagnosis of GPI was the leading cause for referral to psychiatric hospitals until the middle of the 20th century, with an estimated prevalence ranging from 10% to 45% in psychiatric hospitals worldwide.^8^^,^^21^^,^^22^ Because of the time latency between the initial infection and end-stage syphilis, subjects in this group have unsurprisingly had an average older age compared with patients suffering from schizophrenia.

Differences in sex distribution may be explained by the association of syphilis and other sexually transmitted diseases with promiscuity and prostitution, as well as cultural differences in healthcare-seeking behaviors at that time. It is also possible that more aggressive treatment protocols, such as malaria fever therapy, were disproportionately used in male patients, which could explain the predominance of male patients in this cohort.

### Fever course.

According to the literature, the incubation period for *P. vivax* is between 10 and 21 days.^23^^,^^24^ The incubation period in the current study’s patient cohort was considerably shorter at ∼7 days. However, *P. vivax* isolates infecting this patient cohort did not undergo the hepatic developmental stages; therefore, the incubation period constitutes the time of the erythrocytic developmental cycle until symptoms arise. Similarly, dormant liver-stage hypnozoites are found in a natural mosquito-transmitted *P. vivax* infection, which may lead to reemerging disease after weeks or months. In iatrogenic malaria therapy, as applied in this patient population, only blood-stage parasites were inoculated; therefore, the development of liver-stage dormant hypnozoites does not occur. By bypassing the liver stage of the disease, the shorter incubation period until the manifestation of the disease observed in the study groups is therefore explained and depends on the total number of parasites transferred.

Importantly, the study data reveal a shorter periodicity of fever-free intervals between malarial paroxysms compared with the medical literature. Although a 48-hour rhythm of fever episodes is typically reported for *P. vivax* malaria—accounting for natural *P. vivax* infection, with fever peaks on day 1 and day 3—the study data reveal a shorter periodicity, ranging from 30 to 40 hours. Interestingly, the duration of the afebrile period progressively shortened during consecutive fever paroxysms in both study groups.

A possible explanation for this unexpected finding could be the increasing parasite load, leading to a greater induction of immune inflammation in the absence of antimalarial therapy. However, this hypothesis would also apply to natural infections, for which this phenomenon of shortening periodicity has not been observed.

Similarly, increasingly synchronized developmental stages of *P. vivax* are often observed in clinical practice. Because progressive synchronization results in fewer intermittent fever peaks, this would instead result in a longer interval between fever peaks.

With regard to the finding that the overall interval between two fever episodes for individuals who experienced seven fever episodes was shorter than for the overall cohort (i.e., 30.5 hours [IQR: 24–42] versus 39 hours [IQR: 27–45]), a survivor bias cannot be ruled out as a possible explanation for the apparent decrease in the fever-free interval from earlier intervals to the interval between the sixth and seventh fever episodes. Finally, the natural course of *P. vivax* infection was stopped via the administration of antimalarial therapy after a mean of six fever peaks.

The progressive increase in fever peaks with the number of fever periods is consistent with other studies indicating that *P. vivax* infection triggers higher inflammatory host responses than *P. falciparum* and also aggravates clinical symptoms, such as fever and chills, although it features lower parasitemia levels than those observed in* P. falciparum* infections.^17^^,^^25^

Although historical malaria fever therapy has been the subject of retrospective studies before, comparing the study data with those in the available literature is only possible to a limited extent. The reasons for this are manifold. Based on the data from patient files evaluated in various studies, there appears to be no uniform therapeutic standard for malaria fever therapy. Not only did the *Plasmodium* species differ (*P. falciparum, P. vivax, Plasmodium malariae, Plasmodium ovale*), even within a study cohort, but the inoculation dose and route of the malaria pathogen were not uniform. Furthermore, the scientific focus of published studies differed. Collins WE and Jeffrey GM retrospectively examined malaria therapy using induced *P. falciparum* and *P. ovale* infection in a US cohort of neurosyphilis patients in several publications, using both sporozoite- and trophozoite-induced infections, and both primary and secondary infections were considered with regard to the respective immune response and parasitemia.^26^^–^^29^ The focus of McKenzie et al. was on analyzing the correlation between fever and parasitemia and gametocytemia in a historical cohort of US neurosyphilis patients. In these analyses, different inoculated pathogen stages and species were compared with regard to parasitemia and gametocytemia.^30^^,^^31^

### Strengths and limitations.

To the authors’ knowledge, there is no other study from German-speaking countries focused on malaria fever therapy and the detailed description of parasitological and clinical characteristics of iatrogenic *P. vivax* infection in malaria-naïve patients at that time. A strength of the present study’s cohort is the substantial number of patients analyzed compared with previous reports on malaria therapy.^32^^,^^33^ A limitation and weakness of the present study was the high proportion of missing data for laboratory parameters, as well as the absence of any parasitology. Additionally, there was obviously no standardized follow-up monitoring during the treatment period. However, the decrease in hemoglobin levels and erythrocyte counts, as well as the increase in white blood cells, is in line with the understanding of the pathophysiology of *P. vivax* malaria. Severe anemia, particularly in high-transmission areas, is a typical clinical feature in both children and adults.^17^ Severe anemia in *P. vivax* malaria is pathophysiologically explained by the disproportional removal of uninfected red blood cells from the circulation. Compared with only eight red blood cells (RBC) in *P. falciparum*, in *P. vivax* infections, ∼34 uninfected red cells are removed for every infected RBC in the circulation, which may, in addition to the inflammatory response, subsequently result in anemia.^17^^,^^34^

On the basis of the authors’ understanding of *P. vivax* parasite accumulation in the spleen,^35^ data on detailed physical examinations would also have been interesting, particularly with regard to possible spleen enlargement. Unfortunately, there were no corresponding findings reported in the patient records examined.

The comparability with current findings was limited because of laboratory methods no longer used, such as Sahli’s method for hemoglobin quantification. Finally, patients were treated with one or more adjunct treatments, including typhoid inoculation or electroshock therapy. Although these adjunct treatments were most commonly used consecutively, some impact on the observations of clinical data may not be completely ruled out.

## CONCLUSION

This evaluation of historic patient data on malaria fever therapy provides a unique opportunity to study the natural course of infection of untreated *P. vivax* malaria. The historic data reveal surprising clinical features, including increasing fever peaks during the natural course of infection and a progressively shortening periodicity well below the reported 48-hour interval, which puts traditional malariological dogmas into question.
